# Adjuvant Therapy in Resected Adrenocortical Carcinoma: Current Evidence and Patient Selection

**DOI:** 10.3390/cancers18203276

**Published:** 2026-10-11

**Authors:** Lina Pedraza Sanchez, Guilherme Rabinowits, Elio Monsour, Prerna Dogra

**Affiliations:** Department of Head and Neck-Endocrine Oncology, Moffitt Cancer Center, Tampa, FL 33612, USA

**Keywords:** adrenal cancer, adrenal malignancy, mitotane, ADIUVO, ENSAT stage

## Abstract

Adrenocortical carcinoma (ACC) is a rare and aggressive malignancy with recurrence rates of 54–70% following curative-intent resection, even after microscopically complete (R0) surgery. In this review, we summarize the current evidence for adjuvant mitotane, platinum-based chemotherapy, and radiation therapy in resected ACC, and discuss patient selection based on prognostic risk stratification. We also highlight the importance of multidisciplinary management at expert centers, remaining evidence gaps, and future directions including biomarker-driven treatment strategies.

## Simple Summary

Adrenocortical carcinoma is a rare cancer that often recurs after surgery, even when the operation appears to remove the entire tumor. Because the cancer returns in more than half of patients, surgery alone is frequently not enough, and additional treatment is often needed to lower this risk. In this review, we bring together what is currently known about the additional treatments that can be given after surgery, including the medication mitotane, chemotherapy, and radiation. We explain how a patient’s individual risk of relapse can help determine who should receive these additional treatments, and we emphasize that care is best provided by a team of multiple specialists at experienced centers. We also highlight important questions that remain unanswered and areas where further research is needed to guide treatment decisions. By summarizing the current evidence, we aim to offer a useful reference point for clinicians and researchers as the field moves toward treatments tailored to the features of each person’s cancer.

## 1. Introduction

Adrenocortical carcinoma (ACC) is a rare and aggressive malignancy, with an estimated incidence of 1–2 cases per million person-years in the United States. Given its rarity and aggressiveness, therapeutic advances have been slow, and prognosis remains guarded for many patients. Nevertheless, emerging evidence suggests a trend toward improving outcomes in recent decades, largely owing to management at expert centers, completeness of tumor resection, and the increasing use of mitotane and other systemic therapies [1,2].

ACC is characterized by clinical, molecular, and pathological heterogeneity. Approximately 55–68% of tumors are hormonally active, most commonly presenting with cortisol excess (50–80%), followed by androgen excess (40–60%), while aldosterone and estrogen secretion (<5%) are less frequent manifestations [1,3,4]. Transcriptome-based profiling has stratified ACC into two molecular subtypes—C1A (enriched in TP53, CTNNB1, and ZNRF3 mutations; poor prognosis) and C1B (fewer driver mutations; favorable prognosis)—with co-occurring pathway alterations most significantly associated with progression in early-stage disease, underscoring the potential future role of molecular profiling in adjuvant treatment decisions [5,6,7].

In clinical practice, European Network for the Study of Adrenal Tumors (ENSAT) stage at diagnosis remains one of the strongest predictors of outcome, with 5-year overall survival (OS) of 65–82% for stage I, 58–68% for stage II, 35–45% for stage III, and 10–15% for stage IV disease [1,2,3,8]. At diagnosis, approximately 40–65% of patients have locally advanced or metastatic adrenocortical carcinoma (ENSAT stage III–IV), whereas 35–40% present with disease confined to the adrenal gland (ENSAT stage I–II) [1,2,9]. Prognosis is further influenced by tumor proliferative activity, reflected by the Ki-67 index (<10%, 10–19%, ≥20%) or mitotic rate (low grade, ≤20 per 50 HPF [10 mm^2^]; high grade, >20 per 50 HPF [10 mm^2^]), with higher activity associated with shorter recurrence-free survival (RFS) and OS, independent of ACC stage at presentation or completeness of resection [10,11,12].

Surgical resection remains the cornerstone of management for resectable ACC, and achievement of microscopically negative surgical margins (R0) without tumor disruption is one of the most important prognostic determinants [8,13,14]. Open adrenalectomy remains the standard approach for confirmed or highly suspected ACC, as it most reliably achieves an en bloc R0 resection with an intact capsule. When adjacent organs (e.g., kidney, diaphragm, liver, spleen, or pancreatic tail) are directly invaded, the involved portion should be removed en bloc with the tumor to achieve a negative margin. In selected cases, a minimally invasive approach by an experienced surgeon well acquainted with the retroperitoneal space may be considered for relatively small tumors with clear fascial planes and no evidence of extra-adrenal invasion or spread (large-vein tumor thrombus or regional lymphadenopathy) on preoperative imaging. Intraoperatively, should the tumor prove adherent to or invading adjacent structures during a minimally invasive procedure (transabdominal laparoscopic, retroperitoneoscopic, or robotic), the surgeon should have a low threshold to convert to open adrenalectomy to minimize the risk of tumor violation or incomplete resection.

**Figure 1:**
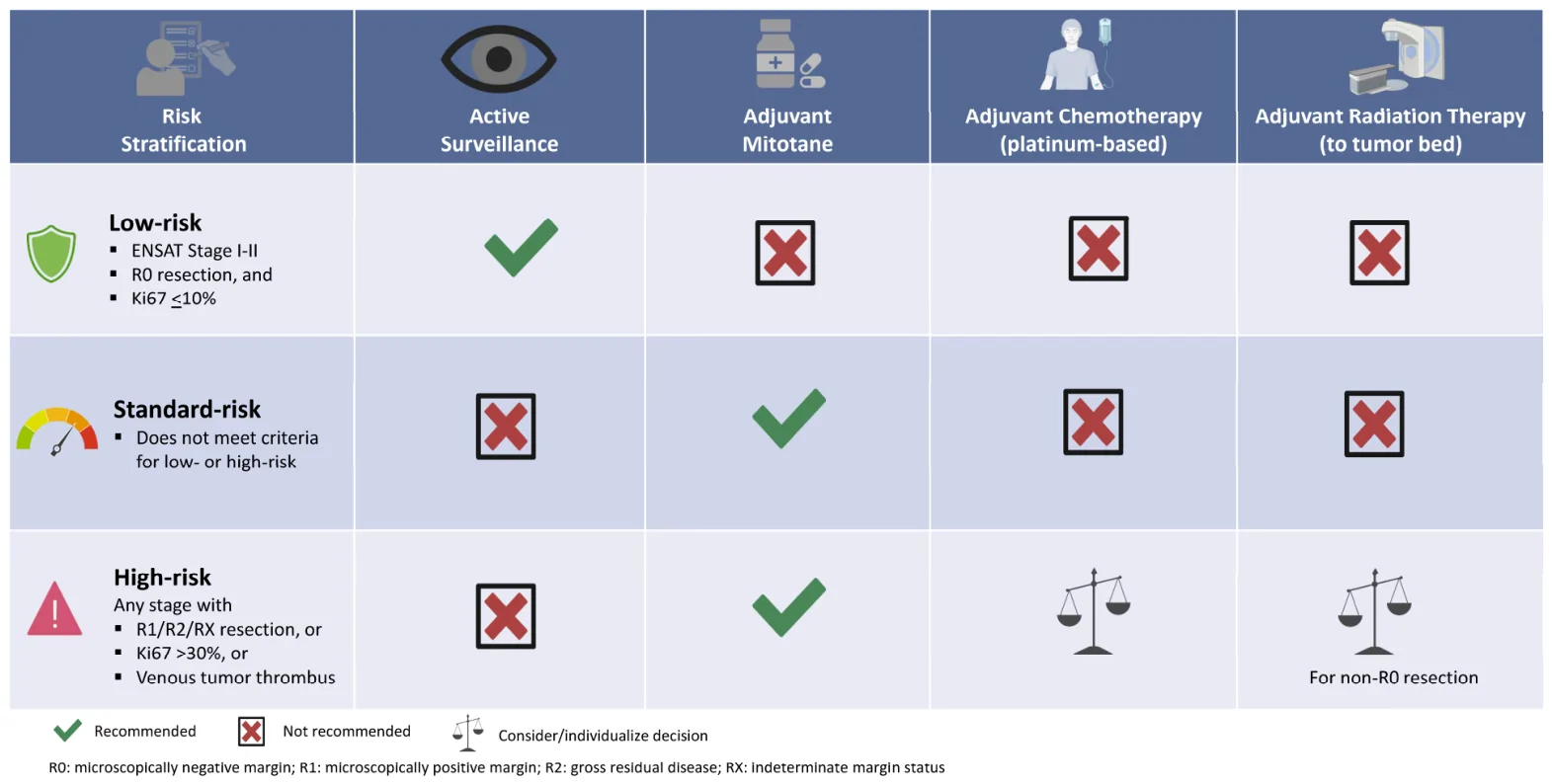
**Practical framework for selecting adjuvant treatment after resection of adrenocortical carcinoma according to recurrence risk based on clinicopathological factors**. Green checkmarks indicate a preferred treatment approach, whereas red X symbols indicate an approach that is not routinely recommended. The grey balance scale indicates that treatment may be considered following individualized assessment of recurrence risk, expected benefit, treatment toxicity, patient preferences, and multidisciplinary discussion. Adjuvant chemotherapy is not routinely recommended for low-risk and standard-risk patients and is therefore not shown as a treatment option for this group. Adjuvant radiation therapy (to tumor bed) is not routinely recommended for microscopically negative margin (R0) resection but should be considered for microscopically positive (R1) or indeterminate margin status (Rx), and for gross residual disease (R2) that cannot be surgically addressed. Surveillance is required across all risk categories, regardless of whether adjuvant treatment is administered.

Even following curative-intent resection, however, recurrence occurs in 54–70% of patients, with risk proportional to the Ki67 index [3,11,13,15]. Given this high recurrence risk, adjuvant therapy plays a pivotal role and should be considered in appropriately selected patients. This review provides a comprehensive overview of adjuvant systemic and radiation therapy for resected ACC, stratified by clinical and pathological risk factors, with a focus on supporting evidence and patient selection (Figure 1; Table 1).

**Table 1:** Summary of key studies informing adjuvant therapy decisions after complete resection of adrenocortical carcinoma.

Study	Study Design	Patient Population	Intervention	Key Outcomes	Limitations
**Adjuvant mitotane**					
Terzolo et al. (*New England Journal of Medicine*, 2007) [16]	Retrospective multicenter study	Radically resected ACC—47 mitotane-treated adults from Italian centers vs. 130 untreated patients from Italian (control group 1) and German centers (control group 2).	Adjuvant mitotane vs. no treatment	Adjuvant mitotane was associated with lower recurrence rate and longer RFS. Median RFS: 42 months in the mitotane group vs. 10 months in control group 1 (*p* < 0.001) and 25 months in control group 2 (*p* = 0.005). Median OS: 110 months in the mitotane group vs. 52 months in control group 1 (*p* = 0.01) and 67 months in control group 2 (*p* = 0.10).	Nonrandomized treatment allocation. Potential selection bias. German controls older and more early stage compared to Italian controls and treated cohort.
Berruti et al. (*Journal of Clinical Endocrinology & Metabolism*, 2017) [17]	Long-term follow-up of the above cohort for an additional 9-years; 3-month landmark exclusion used to mitigate immortal time bias	Radically resected ACC—47 mitotane-treated adults vs. 115 untreated patients from Italian (control group 1) and German centers (control group 2).	Adjuvant mitotane vs. no adjuvant therapy	Benefit of adjuvant mitotane confirmed on a median follow-up of 141 months. Risk of recurrence was higher in both control groups than in the mitotane group: control group 1, HR 2.98 (95% CI 1.75–5.09, *p* < 0.0001); control group 2, HR 2.61 (95% CI 1.56–4.36, *p* < 0.0001) Risk of death was higher in control group 1 (HR 2.03, 95% CI 1.17–3.51, *p* = 0.011) but not control group 2 (HR 1.60, 95% CI 0.94–2.74, *p* = 0.083).	Same cohort as Terzolo 2007 [18]. Therefore, not an independent validation study.
ADIUVO—Terzolo et al. (*Lancet Diabetes & Endocrinology*, 2023) [18]	Multicenter, open-label, phase 3 randomized controlled trial	Resected ACC with low-risk disease (R0 resection, stage I–III, Ki-67 ≤10%)—45 patients treated with mitotane vs. 46 patients received surveillance alone	Adjuvant mitotane vs. surveillance alone.	No survival benefit associated with adjuvant mitotane compared to surveillance alone in low-risk patients. 5-year RFS 79% vs. 75% (HR 0.74, 95% CI 0.30–1.85). 5-year OS 95% vs. 86% (HR 0.46, 95% CI 0.08–1.92)	Underpowered—early closure and low event rate; very few patients with stage III disease; ~40% never reached therapeutic mitotane levels.
Basile et al. (*Journal of Personalized Medicine*, 2021) [19]	Multicenter retrospective study.	154 patients after radical resection; predominantly low risk (~80% stage I–II, median Ki-67 10%).	Adjuvant mitotane treatment duration > 24 months vs. ≤24 months.	Treatment beyond 24 months was not associated with improved RFS after adjuvant mitotane discontinuation (HR 0.894, 95% CI 0.35–2.26, *p* = 0.812)	Retrospective; predominantly low-risk cohort, so limited generalizability to high-risk patients
**Adjuvant chemotherapy**					
Kimpel et al. (*British Journal of Cancer*, 2021) [20]	Multicenter retrospective 1:1 propensity–matched cohort study	Patients with high-risk ACC (median Ki-67 30%; 90% receiving concurrent mitotane)—adjuvant chemotherapy group 31 patients vs. propensity matched 31 patients in non-chemotherapy group	Adjuvant platinum-based chemotherapy vs. no chemotherapy	Prolonged RFS in the adjuvant chemotherapy group (median 17.3 vs. 7.3 months; *p* < 0.001) and improved OS (median not reached in the chemotherapy group vs. 43 months in the non-chemotherapy group, *p* = 0.007).	Retrospective, small sample size; residual confounding possible despite propensity matching.
ADIUVO2 (NCT03583710)	Ongoing international randomized phase III trial	Stage I–III ACC after radical resection (R0, Rx, or R1), Ki-67 >10%, ECOG 0–2; randomization within 90 days of surgery	Adjuvant mitotane alone vs. mitotane + four cycles of cisplatin and etoposide	Designed to address the evidence gap for adjuvant chemotherapy across standard- and high-risk patients	Ongoing; results not yet available.
**Adjuvant radiation therapy (RT)**					
Gharzai et al. (*Journal of Clinical Endocrinology & Metabolism*, 2019) [21]	Single-center retrospective 1:1 propensity-matched cohort study	Stage I–III or oligometastatic stage IV ACC after definitive surgical resection—adjuvant RT group 39 patients vs. propensity matched non-RT group 39 patients	Adjuvant RT vs. none	Adjuvant RT was associated with improved 3-year OS (77.7% vs. 48.6%; *p* = 0.002) and local RFS (59.5% vs. 34.2%; *p* = 0.004)	Single-center, small sample size; outcomes not reported by resection margin status. Residual confounding possible despite propensity matching.
Ginsburg et al. (*Urologic Oncology: Seminars and Original Investigations*, 2021) [22]	NCDB retrospective analysis	Clinically localized ACC treated surgically—adjuvant RT group 259 patients vs. non-RT group 1174 patients	Adjuvant RT vs. none	Adjuvant RT was associated with increased OS in patients with positive surgical margins (HR 0.47, 95% CI 0.35–0.65, *p* < 0.001), large tumor size ≥6 cm (HR 0.69, 95% CI 0.55–0.87, *p* = 0.002), and high-grade disease (HR 0.61, 95% CI 0.37–0.99, *p* = 0.046).	Database study; no recurrence/local control outcomes reported.
Greenspun et al. (*The American Journal of Surgery*, 2025) [23]	NCDB retrospective analysis	Non-metastatic ACC with R0 surgical resection—adjuvant RT group 51 patients vs. non-RT group 337 patients	Adjuvant RT vs. none	Treatment with adjuvant RT was not associated with an OS benefit in patients with ACC who underwent R0 resection. 5-year OS 56% vs. 58%, *p* = 0.54. This was true irrespective of tumor size or high-grade pathology.	Database study; no recurrence/local control outcomes reported.

Studies are grouped by adjuvant modality (mitotane, chemotherapy, and radiation therapy) and ordered chronologically within each group. For each study, the design, patient population, intervention, and principal recurrence-free and/or overall survival outcomes are summarized, together with the main methodological limitations. Except for the ADIUVO trial (Terzolo et al., *Lancet Diabetes & Endocrinology*, 2023) and the ongoing ADIUVO2 trial, all included studies are retrospective. Reported hazard ratios for retrospective studies are therefore adjusted or propensity-matched estimates and should be interpreted accordingly. *Abbreviations*: ACC, adrenocortical carcinoma; CI, confidence interval; ECOG, Eastern Cooperative Oncology Group; HR, hazard ratio; NCDB, National Cancer Database; OS, overall survival; RFS, recurrence-free survival; R0, microscopically negative margin; R1, microscopically positive margin; Rx, indeterminate margin status.

### Case Scenarios

*Patient A:* *47-year-old male with a two-year history of resistant hypertension and hypokalemia is found to have an incidental 8 cm right adrenal mass with unenhanced attenuation of 26 Hounsfield units (HU) on abdominal computed tomography (CT). Physical examination is unremarkable except for generalized obesity. Vital signs include blood pressure 160/80 mm Hg, HR 72 bpm, BMI 36 kg/m^2^. Medications include amlodipine 10 mg once daily, losartan 100 mg daily, hydrochlorothiazide 25 mg daily, and potassium chloride 20 mEq twice daily. Laboratory evaluation reveals morning plasma ACTH 18 pg/mL, serum cortisol 15 mcg/dL, DHEA sulfate 80 mcg/dL, aldosterone < 1 ng/dL, plasma renin activity < 0.1 ng/mL/hr, serum potassium 3.6 mmol/L, and normal plasma fractionated metanephrines. Further work-up shows an elevated 11-deoxycorticosterone level of 150 ng/dL (reference range < 19 ng/dL), normal 11-deoxycortisol, and normal 1 mg dexamethasone suppression test. He is diagnosed with a deoxycorticosterone-secreting indeterminate adrenal mass. He undergoes right adrenalectomy via open approach. Postoperatively, his blood pressure and potassium normalize without need for medications. Final surgical pathology confirms a low-grade adrenocortical carcinoma confined to the adrenal gland (ENSAT stage II), with microscopically negative margins (R0) and Ki-67 of 8%. What is the next best step in the management of this patient?*

*Patient B:* *66-year-old woman with an enlarging left adrenal mass, now measuring 4 cm with an unenhanced attenuation of 32 HU on abdominal CT. She has no past medical history and is not on any medications. Physical examination and vital signs are unremarkable. Hormonal evaluation is unrevealing for pheochromocytoma, autonomous cortisol secretion, and primary aldosteronism. She undergoes laparoscopic left adrenalectomy at a local hospital that reportedly was difficult due to dense periadrenal adhesions. Final surgical pathology confirms a high-grade adrenocortical carcinoma confined to the adrenal gland (ENSAT stage I), with microscopically negative margins (R0) and Ki-67 of 50%. What is the next best step in the management of this patient?*

## 2. Adjuvant Systemic Therapy

In patients with stage I and II ACC, and in most cases with stage III disease, the tumor can be completely resected. However, despite curative-intent surgery, up to two-thirds of patients experience recurrence, highlighting the need for adjuvant therapy. Recurrence risk is largely determined by tumor stage, completeness of resection, and Ki-67 index or tumor grade [8,15]. Based on the prognostication scheme proposed by Fassnacht, Terzolo, and colleagues, resected ACC can be stratified into low-, standard-, and high-risk groups for recurrence [13,24]. ‘Low risk’ includes ENSAT stage I–II disease, R0 resection, and Ki-67 ≤ 10%, while ‘high risk’ is defined by Ki-67 > 30%, incomplete or unknown (R1/R2/Rx) resection status, or venous thrombus. Patients with stage I–III disease who do not meet criteria for either category are classified as ‘standard risk’. By convention, R0 denotes microscopically negative margins, R1 microscopically positive margins, R2 macroscopic residual disease, and Rx indeterminate margin status. Importantly, Ki-67 scoring shows substantial interobserver variability across the measured range, but this variability becomes consequential chiefly near the clinically relevant thresholds (such as the 10% and 30% cutoffs) [25]. Assessment should focus on proliferative hotspots, with experienced (ideally reference-center) pathology review and values near a threshold warrant expert re-review before acting.

### 2.1. Adjuvant Mitotane Monotherapy

Mitotane has been used in the treatment of ACC for more than half a century, receiving FDA approval in 1970, and remaining the only FDA-approved drug for this indication to date. The first strong evidence supporting adjuvant mitotane came from the landmark 2007 retrospective multicenter study, which compared 47 mitotane-treated adult patients with 130 untreated controls from Italian and German centers [16]. Adjuvant mitotane was associated with a lower recurrence rate (49% in the mitotane group vs. 73–91% in the two control groups) and a significantly longer median RFS (42 months in the mitotane group vs. 10–25 months in the two control groups), with benefits being confirmed at the median of the 9-year follow-up [16,17]. Over the past two decades, multiple retrospective studies have replicated the efficacy of adjuvant mitotane, including a recent meta-analysis showing an improved RFS (HR 0.65; *n* = 1845) and OS (HR 0.71; *n* = 1485) in patients who received adjuvant mitotane compared with those who did not [26].

The ADIUVO trial, the first and only randomized controlled trial of adjuvant therapy in ACC, evaluated the role of adjuvant mitotane in patients with low-risk disease. This multicenter, open-label, phase 3 study randomized 91 adult patients meeting criteria for R0 resection, stage I–III disease, and Ki-67 ≤ 10% to receive adjuvant mitotane for 2 years or surveillance alone. At a median follow-up of 48 months, 5-year RFS was not significantly different between the two groups (79% vs. 75%; HR 0.74 [95% CI 0.30–1.85)]. Similarly, no significant difference in the 5-year OS (95% vs. 86%; HR 0.46; 95% CI 0.08–1.92) was observed. While the study was underpowered given early termination due to slow accrual, it provides important evidence that adjuvant mitotane offers limited benefit in patients with low-risk disease. Of note, very few patients in this study had ENSAT stage III disease; therefore, caution is warranted when extrapolating these findings to patients with stage III disease [18].

#### 2.1.1. Mechanism of Action and Pharmacologic Considerations

Mitotane is an analog of the insecticide dichlorodiphenyltrichloroethane (DDT). Although its exact mechanism of action remains incompletely understood, it inhibits adrenal steroidogenesis and exerts direct cytotoxic effects on adrenocortical cells. It is highly lipophilic, resulting in a prolonged and variable half-life (18–159 days) [27]. Additionally, it is a potent CYP3A4 inducer, which has important clinical implications. First, mitotane accelerates the metabolism of exogenous glucocorticoids and, in combination with increased hepatic synthesis of cortisol-binding globulin, renders standard hydrocortisone replacement doses (15–25 mg/day) insufficient; most patients require 50–60 mg/day, and some even higher doses [28]. Mineralocorticoid replacement is also required in approximately 30% of patients receiving chronic mitotane therapy, with fludrocortisone dosing guided by clinical assessment, blood pressure, serum potassium and plasma renin activity. Second, as a strong CYP3A4 inducer, mitotane can substantially reduce the efficacy of numerous co-administered medications, including hormonal contraceptives, warfarin, and azoles, necessitating careful medication review, dose adjustments, and the use of barrier contraception in women of childbearing age. Furthermore, mitotane inhibits 5α-reductase activity, which, in combination with increased sex hormone–binding globulin synthesis, may contribute to hypogonadal symptoms in men and can complicate testosterone replacement by reducing bioavailable testosterone [28].

#### 2.1.2. Dose Titration

There is no fixed dose or standardized titration protocol for mitotane. Mitotane is started at a low dose, typically 1.0–1.5 g/day, and the dose titrated to 3.0–6.0 g/day to maximize patient tolerance. In a prospective comparison of two titration regimens in 32 patients with advanced ACC, low-dose (1 g on day 1, increasing by 0.5 g every 3–4 days to a total of 3.0–4.0 g/day) and high-dose (1.5 g on day 1, 3.0 g on day 2, 4.5 g on day 3, and 6.0 g on day 4), the median maximum plasma levels achieved were not significantly different at 12 weeks, although cumulative mitotane exposure was higher in the high-dose group [29]. These findings support individualized titration guided by patient tolerability, comorbidities, and early plasma concentration monitoring rather than a uniform fixed-dose strategy.

#### 2.1.3. Therapeutic Levels and Monitoring

The commonly accepted therapeutic range for mitotane is 14–20 mg/L, established decades ago on the basis of small retrospective studies suggesting that plasma concentrations ≥ 14 mg/L were associated with improved disease control, while levels exceeding 15–20 mg/L were linked to increased neurotoxicity [30,31]. These thresholds have persisted in clinical practice largely unchallenged, despite never having been validated in prospective trials [32]. In a retrospective multicenter study of 110 adjuvant-treated patients, multivariate analysis showed that the Ki67 index and time to achieve the target range of plasma mitotane were independent predictors of RFS [33]. Nevertheless, objective tumor responses have also been reported at concentrations below the traditional therapeutic range [34]. In the retrospective analysis of 91 patients with advanced ACC, the 14 mg/L cutoff had a sensitivity of only 65% and specificity of 69% for objective response, with over one-third of patients achieving response at subtherapeutic levels—underscoring the limitations of a fixed concentration threshold [35]. Furthermore, achieving and maintaining therapeutic range remains challenging in routine practice due to patient tolerance and inter- and intraindividual variability. Even in the ADIUVO trial—conducted at expert centers with protocolized monitoring—approximately 40% of patients failed to achieve therapeutic concentration [18]. Considering all of the above, therapeutic drug monitoring remains essential; however, the 14 mg/L threshold is best regarded as a practical benchmark rather than a definitive cutoff, and failure to reach it should not be equated with treatment futility [32].

Mitotane levels are generally monitored every 3–4 weeks during dose escalation and every 2–3 months once stable. Monitoring should also include liver function, lipid profile, thyroid function, adequacy of glucocorticoid replacement, neurologic assessment, gastrointestinal tolerance, and potential drug–drug interactions. Adverse effects are common—including gastrointestinal symptoms, fatigue, cognitive impairment, hepatotoxicity, dyslipidemia, adrenal insufficiency, hypothyroidism, hypogonadism, and neurotoxicity—but are generally manageable with proactive monitoring, symptomatic treatment, and dose adjustment; in the ADIUVO trial, approximately 19% of patients discontinued treatment [18,36,37].

#### 2.1.4. Predictors of Mitotane Response

Response to mitotane is highly variable, and no validated biomarkers currently predict attainment of therapeutic concentrations or clinical response. Pharmacogenomic variability may contribute to these differences. In a multicenter cohort of 182 patients with ACC, those carrying the CYP2W1*6 polymorphism achieved therapeutic mitotane concentrations (>14 mg/L) less frequently than wild-type carriers (51.7% vs. 76.2%, *p* = 0.051), demonstrated shorter time to progression (HR 2.10, *p* = 0.019) and lower disease control rates (*p* = 0.008) [38]. In contrast, patients with the CYP2B6*6 polymorphism more frequently achieved therapeutic mitotane levels compared with wild-type carriers (55.0% vs. 28.2%, *p* = 0.016). Furthermore, the combined CYP2W1*6 wild-type and CYP2B6*6 genotype was associated with the highest rate of achieving therapeutic mitotane concentrations (60.6%, *p* = 0.034), suggesting a potential role for pharmacogenomic profiling in predicting individual response to mitotane. At the tumor level, several molecular features have been linked to mitotane sensitivity. High RRM1 (ribonucleotide reductase large subunit 1) expression has been associated with mitotane resistance and worse disease-free survival, with the benefit of adjuvant mitotane confined to patients with low RRM1 expression [39]. Analysis of patient-derived cell cultures has also implicated specific genomic alterations: ZNRF3 alterations were linked to mitotane resistance, whereas CTNNB1 mutations were associated with sensitivity [40]. Transcriptomic profiling has shown lipid metabolism pathways to be upregulated in responder tumors, suggesting that mitotane sensitivity may depend on the tumor’s lipid metabolic machinery [40]. However, these findings are derived from retrospective cohorts or preclinical models, their predictive power remains limited, and none have been prospectively validated.

#### 2.1.5. Duration of Therapy

The optimal duration of adjuvant mitotane therapy remains uncertain [16,17]. The recommendation to continue adjuvant mitotane for at least 2 years is based primarily on a multicenter retrospective study in which treatment beyond 24 months was not associated with improved recurrence-free or overall survival among the 154 patients who underwent radical resection. However, the study population was predominantly low risk, with approximately 80% of patients having stage I–II disease and a median Ki-67 index of 10%, limiting generalizability to higher-risk populations [19]. The ADIUVO trial, which similarly enrolled only patients with low-risk ACC (R0 resection, Ki-67 ≤ 10%), and adopted a treatment duration of 2 years, did not demonstrate a meaningful benefit over surveillance, questioning the role of adjuvant therapy in this population altogether [18]. Conversely, no study has specifically evaluated the optimal duration of adjuvant mitotane therapy in patients at high risk of recurrence, for whom the potential benefit of treatment is the greatest. In this population, treatment beyond 2 years and up to 5 years should be considered in those who maintain an acceptable tolerability and side-effect profile.

### 2.2. Adjuvant Cytotoxic Chemotherapy

Although adjuvant mitotane improves RFS and OS following curative-intent resection, up to 50% of patients experience recurrence despite treatment. This risk is particularly elevated in patients with adverse prognostic markers—including higher Ki-67 index, R1/R2/Rx resection, and advanced ENSAT stage [8]. The strongest supporting evidence for adjuvant chemotherapy comes from a multicenter retrospective propensity score–matched cohort study, which evaluated adjuvant platinum-based chemotherapy plus mitotane in 31 patients with high-risk ACC (median Ki-67 of 30%; 90% receiving concurrent mitotane) [20]. The addition of platinum-based chemotherapy was associated with significantly prolonged RFS (median 17.3 vs. 7.3 months; adjusted HR 0.19, 95% CI 0.09–0.42, *p* < 0.001; propensity score–matched HR 0.45, 95% CI 0.29–0.89, *p* = 0.021) and improved OS (HR 0.25, 95% CI 0.09–0.69, *p* = 0.007). However, this study is limited by its retrospective design and small sample size. To address this evidence gap, the ADIUVO2 trial (NCT03583710) is an ongoing international, randomized, phase III study comparing adjuvant mitotane alone versus mitotane combined with four cycles of cisplatin and etoposide. The trial enrolls patients with stage I–III ACC after radical resection (R0, Rx, or R1) with Ki-67 > 10% and ECOG performance status 0–2, with randomization required within 90 days of surgery—thus encompassing both standard- and high-risk patients. Enrollment of all suitable patients is strongly encouraged. Per expert consensus, when enrollment is not possible, four cycles of cisplatin and etoposide in addition to mitotane should be considered in patients at high risk of recurrence (Ki-67 > 30%, non-R0 resection, venous tumor thrombus) [12,24].

## 3. Adjuvant Radiation Therapy (RT)

Locoregional recurrence is common following resection of ACC [13,15,41]. In a multicenter US analysis of 180 patients, 64.4% recurred, of whom 36.3% had locoregional-only and 18.6% had combined locoregional and distant recurrence, with a median time to recurrence of 18.8 months. In a separate analysis from the same collaborative dataset, positive surgical margins were strongly associated with worse outcomes, with 5-year RFS of 13.8% for R1 versus 30.3% for R0 resection (*p* = 0.03) [13]. In another single-institution study of 354 patients from the University of Michigan, more than two-thirds recurred at a median recurrence-free interval of 11 months [28]. Locoregional recurrence was prominent, with tumor bed or peritoneal cavity involvement in approximately half of recurrences. Surgical approach influenced recurrence patterns: laparoscopic resection was associated with higher rates of positive margins (29% vs. 21%, *p* = 0.045) and peritoneal cavity recurrence (31% vs. 16%, *p* = 0.002) compared with open resection [15].

The role of adjuvant RT in ACC remains supported primarily by small retrospective or administrative database studies. The National Comprehensive Cancer Network guidelines recommend considering RT to the surgical bed in patients with high-risk features for local recurrence (positive margins, Ki-67 > 10%, rupture of the tumor capsule, large tumor size, or high grade) based on low-quality evidence [42]. A propensity-matched analysis of 39 RT-treated patients showed improvements in 3-year OS (77.7% vs. 48.6%; HR 3.59 in non-RT, *p* = 0.002) and local RFS (59.5% vs. 34.2%; HR 2.67 in non-RT, *p* = 0.004), without stratifying by margin status or other clinicopathologic risk factors [21]. A National Cancer Database (NCDB) analysis of 259 RT-treated patients demonstrated an OS benefit, most pronounced in patients with positive margins (HR 0.47) [22]. A 2025 meta-analysis reported significant benefits for adjuvant RT in stage I–III disease for both OS (HR 0.68) and RFS (HR 0.71) [26]. Conversely, a recent NCDB analysis of 51 RT-treated patients after R0 resection found no survival benefit [23]. Collectively, the available data suggest that the benefit of adjuvant RT may be most apparent in patients with positive margins, though definitive conclusions are limited by the retrospective nature of all studies, the inability of population-based administrative databases to capture recurrence data, the lack of important information on Ki-67, hormonal secretion, RT dose and indication, and the absence of stratification by margin status with local recurrence-free survival as the primary endpoint—arguably the most clinically relevant measure for adjuvant RT.

In practice, at most expert centers, adjuvant RT is primarily directed at patients with R1 or Rx margins. Notably, at our center, Rx status is also assigned when the initial resection was performed via a minimally invasive approach at an outside institution, where the adequacy of oncologic margins and the integrity of the tumor capsule cannot be reliably confirmed. While concurrent chemoradiation is well established in other malignancies, combining retroperitoneal radiation with platinum-based chemotherapy in ACC is limited not only by the lack of supporting evidence but also by concern for additive toxicity. Accordingly, decisions regarding the timing and sequencing of RT with systemic therapy should be individualized through multidisciplinary discussion.

### Case Scenarios Continued

*Patient A: Based on the risk-adapted approach, Patient A was stratified to low risk of recurrence. Adjuvant therapy was not indicated. He is on active surveillance with CT of the thorax, abdomen, and pelvis every 3 months for 2 years, followed by every 6 to 12 months for a total of 10 years if no recurrence is detected. He remains disease-free at 18 months of follow-up.*

*Patient B: Even though surgical histopathology showed microscopically negative margins (R0), tumor capsule integrity could not be confirmed, leaving uncertainty about the completeness of resection (Rx). Her ACC was classified as high risk based on a Ki-67 of 50%, despite ENSAT stage I disease, supporting adjuvant mitotane. Concern about capsule integrity contributed to the multidisciplinary decision to add tumor-bed radiation therapy. She underwent adjuvant intensity-modulated radiation therapy to the left tumor bed for a total dose of 54 Gy, started on adjuvant mitotane therapy titrated to 4.5 g/day with good tolerance, and underwent imaging surveillance with CT of the thorax, abdomen, and pelvis every 3 months. Unfortunately, she developed distant recurrence with pulmonary metastases after a recurrence-free interval of 8 months, and escalation of systemic therapy is planned. This case illustrates how tumor biology and operative findings can influence adjuvant decisions beyond stage alone.*

## 4. Surveillance

Structured surveillance is an essential component of care following ACC resection, whether or not adjuvant therapy is given. Median recurrence intervals of 11 and 18.8 months in single-center and multicenter cohorts, respectively, support frequent imaging during the initial postoperative years [15,41]. Cross-sectional imaging of the chest, abdomen, and pelvis (chest CT with or without contrast, plus CT or MRI of the abdomen and pelvis with contrast) is recommended every 3 months for 2 years, then every 3–6 months for a further 3 years, with less frequent, individualized follow-up thereafter in disease-free patients [42]. Surveillance should also include biochemical assessment, with hormonal testing guided by the tumor’s preoperative secretory profile so that any originally secreted hormones or steroid markers can be tracked as early signals of recurrence; this biochemical testing can follow the same schedule as imaging.

## 5. Multidisciplinary Management

The foundation of ACC management is a dedicated multidisciplinary team, ideally based at a high-volume referral center, as this coordinated approach has been linked to improved oncologic outcomes [1]. Such a team should bring together the complementary expertise of endocrinologists, surgeons, diagnostic radiologists, pathologists, medical oncologists, radiation oncologists, and interventional radiologists, allowing a collaborative discussion regarding tumor resectability, surgical approach, and the sequencing of neoadjuvant, adjuvant, and locoregional therapies. Within this framework, treatment goals are individualized for each patient, balancing the pursuit of longevity against the morbidity of extensive surgery and systemic therapy so that every decision is a deliberate assessment of anticipated benefit, treatment-related risk, and quality of life—a goal that can only be reached by a collaborative team equipped with the necessary resources and experienced in both delivering these therapies and managing their adverse effects.

## 6. Future Perspectives

A major gap in the adjuvant management of resected ACC is the inability to reliably predict which patients will recur and which will respond to mitotane. Current risk stratification relies on clinical and pathological factors, but these incompletely capture the biological heterogeneity of ACC. Molecular classification offers a path forward: pan-genomic profiling has identified specific molecular biomarkers that are strong independent predictors of recurrence after complete resection—adding significant prognostic value beyond ENSAT stage and Ki-67 [43]. Emerging evidence demonstrates that focused NGS panels can translate these findings into clinically actionable tools, particularly in early-stage disease [6,44].

**Figure 2:**
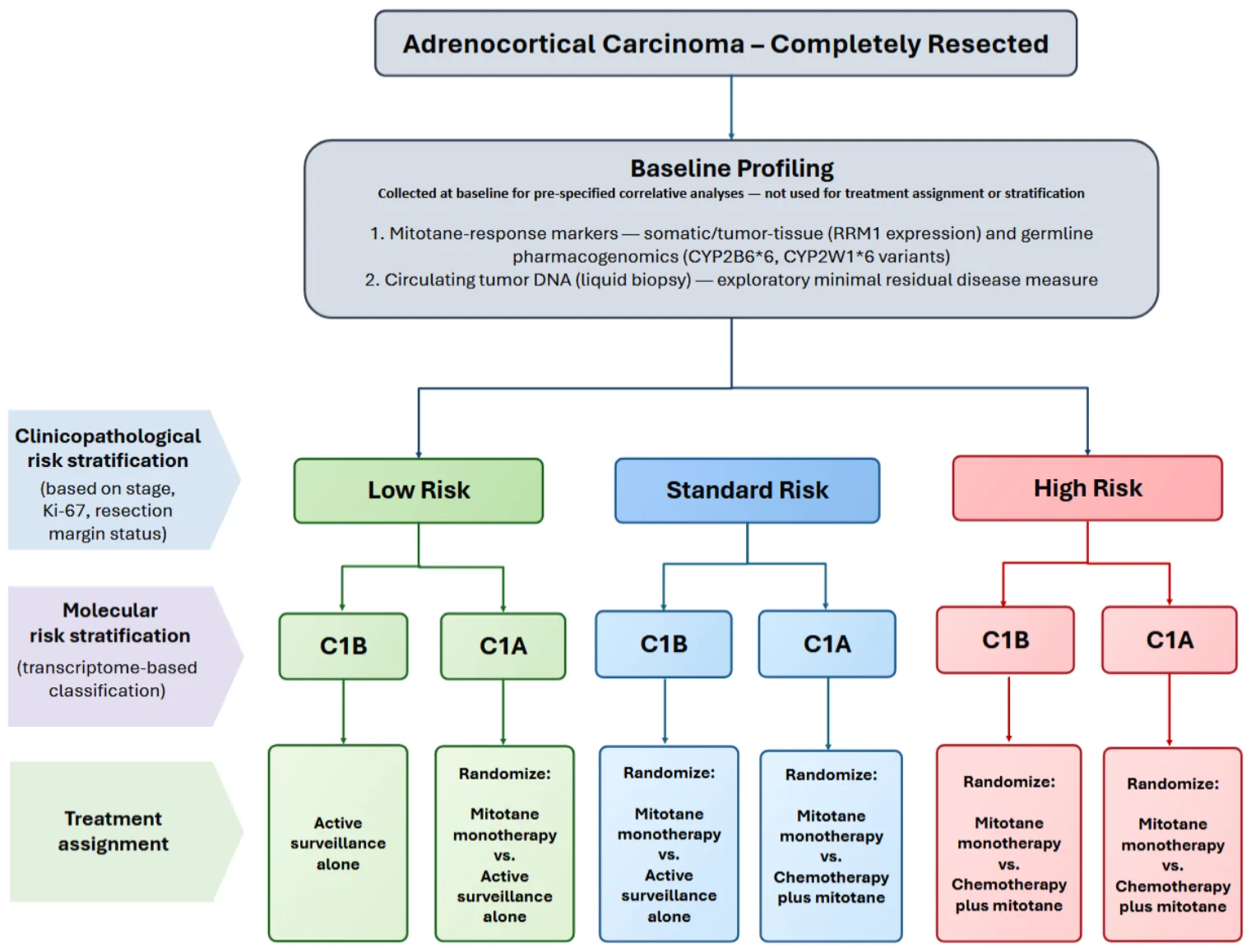
**Hypothetical model for a prospective biomarker-integrated adjuvant trial in resected adrenocortical carcinoma (ACC)**. Because current clinicopathological risk stratification does not fully capture recurrence risk—some low-risk patients recur while some standard- and high-risk patients never do—we hypothesize that adding transcriptome-based molecular risk stratification refines prognostication and improves selection for adjuvant therapy. In the proposed model, all patients with resected ACC are stratified first by clinicopathological risk and then by molecular risk and are assigned to adjuvant mitotane monotherapy, chemotherapy plus mitotane, or surveillance accordingly. *Baseline profiling*: Mitotane-response markers (somatic/tumor-tissue expression and germline pharmacogenomics) and postoperative circulating tumor DNA (as an exploratory minimal-residual-disease measure) are collected at baseline for pre-specified correlative analyses only. These variables are not used for treatment assignment or stratification in this model, as none is prospectively validated as predictive in ACC. *Clinicopathological risk stratification*: low risk = stage I–II with microscopically negative resection margins (R0), and Ki-67 ≤ 10%; high risk = stage I–III with incomplete or unknown resection margin status (R1/Rx) or Ki-67 > 30%; standard risk comprises resected tumors meeting neither low-risk nor high-risk criterion. *Molecular risk stratification*: Transcriptome-based classification defines C1A (poor prognosis) vs. C1B (favorable prognosis), as originally described by de Reyniès et al. (*Journal of Clinical Oncology*, 2009) [7] and retained as the transcriptome component of the integrated genomic classification of Assié et al. (*Nature Genetics*, 2014) [5]. Molecular class is used for treatment assignments in the low- and standard-risk clinicopathological tiers; in the high-risk tier, where both classes receive identical arms, it serves only as a stratification/analysis variable.

These advances have the potential to reshape adjuvant decision-making across the risk spectrum, allowing tailored escalation or omission of adjuvant therapy based on tumor biology. Prospective trials integrating molecular profiling into adjuvant treatment allocation represent an important next step toward precision-guided management of adrenocortical carcinoma. Figure 2. From a therapeutic standpoint, predicting mitotane response will require multi-biomarker approaches incorporating tumor-level markers such as RRM1 expression and genomic alterations, alongside germline pharmacogenomic profiling [38,39,40]. If validated, such composite panels could inform dosing strategy—sparing sensitive patients from dose-related toxicity while redirecting resistant patients to alternative therapies earlier. Complementing tissue-based analyses, liquid biopsy strategies—including urine or plasma steroid metabolomics, circulating microRNAs, and cell-free DNA—may enable molecular monitoring of treatment response and earlier detection of recurrence [45,46,47].

## 7. Conclusions

In summary, current evidence supports a risk-adapted approach to adjuvant therapy for resected ACC. Low-risk patients (ENSAT stage I–II, R0, and Ki-67 ≤ 10%) can be managed with active surveillance. Whereas in standard- and high-risk patients, adjuvant mitotane should be strongly considered. In those with high-risk features—including Ki-67 > 30%, non-R0 resection, or venous tumor thrombus—consideration should also be given to adjuvant platinum-based chemotherapy. In patients with R1 or Rx margins, adjuvant radiation therapy to the tumor bed should be considered to reduce local recurrence risk. The sequencing and combination of RT with systemic therapy should be individualized through multidisciplinary discussion. Given the rarity and complexity of ACC, management at expert centers with multidisciplinary teams experienced in this disease is essential to optimize outcomes. Continued enrollment in prospective clinical trials remains critical to refining treatment strategies and improving survival for patients with ACC at all stages of disease. Ultimately, integrating multi-omic tools into prospective clinical trials will be critical to moving toward a precision-based treatment approach.

## Data Availability

No new data were created or analyzed in this study. Data sharing is not applicable to this article.
